# A Lepers' Home

**Published:** 1898-01-08

**Authors:** 


### A Lepers' Home.

### THE COAST HOSPITAL, LITTLE BAY, SYDNEY.

Many have found the road to the hospital anything but a pleasant drive, for the joltings caused by the ruts and holes play pitch and toss with one's bones, and a finger-post pointing to the cemetery is suggestive and not cheering. One wonders if the vehicle does not sometimes turn aside there, but consoles one's self with the reflection that the jolting may possibly be good for a sluggish liver. The road ends amid a broad expanse of scrub and sand, over which in the spring Nature flings a veil of verdure enamelled with flowers. In front the bright blue waters of the broad Pacific roll in on the rocky shore, flinging their white foam high up against the frowning cliffs, and little rainbows glint and vanish with the silver spray. As one inhales the fresh ozone one deems they wisely chose who placed a sanatorium on such a site. The numerous separate pavilions of the large hospital nestle in the hollows or crown the heights which surround a pretty little bay. So scattered are the buildings that they cannot all be taken in at a glance. In a depression on the extreme left of the little township, for such it appears, there are two rows of small detached cottages facing each other, built, like all the rest of the hospital, of painted corrugated iron. Each tenement has before it a railed-in enclosure with an entrance gate. In some the little space is bright with flowers or green with vegetables, but others are weedy, forlorn, and desolate. The inmates of these dwellings are a people who live alone and separate from their fellows, for though they have a considerable extent of bush and seashore at their disposal they are debarred from entering the hospital proper. Each inmate has for private use two rooms—a bed-room and a sitting-room—with washhouse, bath-room, &c., at the back. The rooms are well furnished. The neat bedsteads with sanitary wire mattresses look nice and comfortable with their white mosquito curtains; the bedsteads are often loaded with two or more mattresses, quantity and comfort being looked on as synonymous. The floors are covered with linoleum and loose strips of carpet and rugs. The walls are hung with pictures, generally marked by a great absence of arrangement and symmetry; sofas and easy chairs are provided *ad lib.*, cupboards and tables are loaded with vases, nick-nacks, and photos given by Government or by various officials of the place, till some of the rooms are veritable curiosity shops. There is also a large and lofty social hall, containing a piano, a harmonium, and a very good billiard table, and other games. Such is the Lepers' Home, for so it should be termed rather than a hospital. Until very lately a cure was regarded as utterly hopeless, and the treatment was merely palliative, but now high hopes are entertained that curative measures have been discovered. Only a qualified bacteriologist could describe them, and it is as yet premature to pronounce a definite opinion on their value.

